# Administration of intravenous antibiotics in patients with open fractures is dependent on emergency room triaging

**DOI:** 10.1371/journal.pone.0202013

**Published:** 2018-08-14

**Authors:** Katharine D. Harper, Courtney Quinn, Joshua Eccles, Frederick Ramsey, Saqib Rehman

**Affiliations:** 1 Department of Orthopaedic Surgery and Sports Medicine, Temple University Hospital, Philadelphia PA, United States of America; 2 Lewis Katz School of Medicine at Temple University, Temple University School of Medicine, Philadelphia, PA, United States of America; 3 Lewis Katz School of Medicine at Temple University, Department of Clinical Sciences, Temple University School of Medicine, Philadelphia, PA, United States of America; Flinders University, AUSTRALIA

## Abstract

**Background:**

Recent literature has demonstrated that emergent administration of antibiotics is perhaps more critical than even emergent debridement. Most recent studies recommend patients receive antibiotics no later than 1 hour after injury to prevent infection. The objective of this study is to evaluate the time to antibiotic administration after patients with open fractures arrive to a trauma center depending on triaging team.

**Methods:**

A retrospective study at a level 1 Trauma center from January 2013 to March 2015 where 117 patients with open fractures were evaluated. Patients who presented with open fractures of the extremities or pelvis were considered. Subjects were identified using Current Procedural Terminology (CPT) codes. Patients aged 18 and older were analyzed for Gustilo type, antibiotics administered in the emergency room (ER), presence of an antibiotic allergy, post-operative antibiotic regimen and number of debridements, among others. Additionally, whether a patient was triaged by ER doctors or trauma surgeons (and made a trauma activation) was evaluated. Outcome measurements included time to intravenous (IV) antibiotic administration and time to surgical debridement.

**Results:**

Patients received IV cefazolin a median of 17 minutes after arrival. Eighty-five patients who were made trauma activations received cefazolin 14 minutes after arrival while 24 non-trauma patients received cefazolin 53 minutes after arrival (p = <0.0001). The median time to gentamicin administration for all patients was 180 minutes. Patients not upgraded to a trauma received gentamicin 263 minutes after arrival, while patients upgraded received gentamicin 176 minutes after arrival. There was no statistically significant difference between the timing to cefazolin or gentamicin based on Gustilo type.

**Conclusions:**

Overall, patients that arrive at our institution with open fractures receive IV cefazolin significantly faster when trauma surgeons evaluate the patient. Additionally, delays in gentamicin administration are demonstrated in both triaging groups. This is due to the fact that cefazolin is stocked in the hospital ER, while gentamicin is commonly not due to weight-based dosing requirements precluding a standard dose. Improvements can be made to antibiotic administration of non-trauma patients and those requiring gentamicin via improved education and awareness of open fractures.

## Introduction

Open fractures usually result from high-energy traumatic mechanisms when bone or fragments of bone penetrate the skin and are exposed to the external environment [1]. Classification was developed for open fractures based on the severity of soft tissue injury. Type I injuries being an open fracture with a less than 1cm puncture wound or simple fracture pattern, type II as a fracture with a 1–10cm laceration with moderate soft tissue damage or moderately complex fracture pattern and type III as a fracture with extensive soft tissue damage or highly comminuted fracture pattern (segmental, crush) [1,2]. Revision of this classification system by Gustilo et al subdivided type III open fractures into type IIIA, corresponding to adequate soft tissue coverage of a fractured bone, type IIIB as fractures with extensive injury requiring full thickness soft tissue procedures for coverage and type IIIC as open fractures associated with arterial injury requiring repair [3]. Risks of developing a fracture-related infection include fracture location, fracture severity, timing to antibiotic administration, and time to operative management [4,5,6,7]. Harris et al found that the most common complication from severe limb-threatening lower extremity trauma, including Gustilo type IIIB, IIIC and selected type IIIA fractures, was wound infection [8]. Empirically, type I fractures correlate with a 0–2% clinical rate of infection, type II fractures correlate with a 2–10% rate of infection and type III fractures correlate with a 10–50% rate of infection [1,4,5,6,9].

Management of such injuries includes adherence to Advanced Trauma Life Support guidelines, wound coverage with a dressing soaked in sterile saline, fracture stabilization, prophylactic tetanus toxoid administration, therapeutic antibiotic administration, and wound debridement [1,10,11]. Antibiotics should be administered as soon as possible following the injury with the Gustilo classification system of open fractures dictating the specific class and duration of antibiotic [10].

Current Eastern Association for the Surgery of Trauma (EAST, USA) guidelines state that antibiotic coverage for Gram-positive bacteria (e.g. cefazolin) should be started as quickly as possible after injury with concomitant Gram-negative coverage (e.g. aminoglycosides) for more severe open fractures (type III) [10]. This initial course of antibiotics has been shown to significantly lower the risk of infection from open fractures in accordance with proper wound management [1,7,10,11,12,13]. In a study of 137 patients with type III open tibia fractures, increased time to antibiotic administration correlated to a rise in infection rate, specifically an infection rate of 6.8% for antibiotics administered within the first hour after injury, as compared to 18% for antibiotics between 60 and 90 minutes and 27.9% for antibiotics longer than 90 minutes [14]. Though antibiotics should be given as soon as possible after injury, the duration of prophylactic antibiotic therapy is not related to the risk of infection [12].

Current protocol at our institution aims to give antibiotics as soon as possible following patient arrival with cefazolin (1g) given for type I and II fractures, and concomitant cefazolin and gentamicin (5mg/kg body weight) for type III fractures. For open fractures of any type with soil contamination, penicillin (3 million units) is given every 4 hours. Other institutions have a similar goal, but some have reported that the actual timing is not as optimal as it can be. Specifically, a study by Lack et al showed that despite improved transportation times, only a minority of patients received antibiotics within an hour of injury, and in fact only 50% of patients arrived to the hospital within 1 hour of injury [14]. This points to how vital it is for patients to get antibiotics immediately upon arrival. We have anecdotally noted that at our institution, despite our intentions, antibiotic administration is not always done as quickly as we would like in these circumstances. The purpose of this retrospective study is to determine the timing to intravenous (IV) antibiotic administration to patients with open fractures presenting to our level 1 trauma center and to identify any possible reasons for delay.

## Method and materials

The Temple University Hospital Institutional Review Board approval was obtained prior to accessing identifying patient data, and the need for consent was waived due to the retrospective nature of the study. A retrospective, observational study was performed at our level 1 trauma center over a two-year period from January 1, 2013 to March 31, 2015. All adult patients who presented to the Emergency Department with open fractures of the extremities and/or pelvis were considered for this study. Subjects were identified using our departmental database by searching both procedures and diagnoses for open fractures as well as cross referencing with patients treated at our institution using the Current Procedural Terminology (CPT) codes 11010, 11011, and 11012 (Debridement including removal of foreign material associated with open fractures). Only those patients age 18 and older were analyzed with the following items being obtained from the medical record: age, gender, BMI, transportation method to the hospital, fracture location, Gustilo type, side of injury, presence of poly-trauma (>1 long bone or pelvic fracture, head injury, chest injury, or abdominal injury), any other associated orthopaedic or non-orthopaedic injuries, mechanism of injury, antibiotics administered in the emergency department, the presence of a penicillin or cephalosporin allergy requiring use of an alternative antibiotic, post-operative antibiotic regimen ordered, the number of repeat debridements (if indicated), the need for and type of soft tissue coverage, and whether there was a reported infection at the operative site. We also analyzed which patients were upgraded to a “trauma activation,” meaning the patient was formally evaluated by the general surgery trauma team in the trauma bay as opposed to being cared for by the emergency department physicians.

The time after arrival to administration of cefazolin, gentamicin (if applicable), or penicillin (if applicable), as well as the time to surgical debridement were calculated based on the patient’s arrival time to the Emergency Department (defined as the time they arrived to the triage area) and the documented time the specific antibiotic was given and the documented operative start time, respectively. The transportation time to the hospital was calculated based on emergency medical services (EMS) records. No patients in our data set arrived in private vehicles. Exclusions for this study include undocumented timing of antibiotic administration, patient transfers from non-affiliated hospitals, patients less than 18 years old and patients who presented more than 24 hours after injury. Patients allergic to antibiotics given as part of the standard protocol were included with the appropriate recommended alternative antibiotic as a surrogate for cefazolin. Patients transferred from our hospital’s satellite emergency room (ER) were included if the original emergency department record was available. Patients with fractures from low velocity gunshot injuries were considered Gustilo type 1 injuries unless specified otherwise by the treating physicians.

Antibiotics were dosed according to standard practice of care. Cefazolin was administered as a standard 1gram dose every 8 hours x 3 doses. Gentamicin dose is a weight-based dose with renal clearance taken into consideration for dose timing. Patients receive 5mg/kg now and then every 24 hours x 2 doses if renal function normal (creatinine clearance >/ = 60 mL/min), 5 mg/kg now and then every 36 hours x 1 dose if creatinine clearance between 40–59 mL/min and 3mg/kg once at time of injury if creatinine clearance < / = 39 mL/min.

### Statistical analysis

Descriptive statistics were calculated for both categorical and continuous variables. Data was presented as mean with standard deviation, minimum, maximum, median, and percentages. Select variables were then analyzed using parametric (t-test and Analysis of Variance) and non-parametric (Wilcoxon and Kruskal-Wallis) testing for the timing to administration of cefazolin & gentamicin, and gender & Gustilo type, respectively. Statistical significance was defined as a probability value (p-value) less than 0.05. P-values that exceeded 0.05 were still considered or evaluated. Although both mean and median were reported for this study, the presence of outliers could skew the data with mean calculations. Therefore, the median values were used as the most representative descriptor of central tendency. Data were analyzed using Statistical Analysis System 9.4 (SAS Institute, Cary NC, USA).

## Results

The final cohort consisted of 117 patients with open fractures following exclusions for undocumented timing of antibiotic administration (1), patient transfers from non-affiliated hospitals (11), patients less than 18 years old (1) and patients who presented more than 24 hours after the injury (1). The 117 patients consisted of 29 females (24.8%) and 88 males (75.2%) with 53 patients age 18–29 (45.3%), 27 patients age 30–39 (23.1%), 19 patients age 40–49 (16.2%) and 18 patients age 50 or older (15.4%).

Out of the 117 patients included, 36 (30.8%) had an open fracture of the upper extremity while 81 (69.2%) had an open fracture of the lower extremity. Based on the treating physicians’ Gustilo-Anderson classification of open fractures, 53 (45.3%) were type I; 25 (21.4%) were type II; 25 (21.4%) were type IIIa; 10 (8.5%) were type IIIb; and 4 (3.4%) were type IIIc. The number of patients that were upgraded to trauma surgery activation was 91 (77.8%), with 17 (14.5%) having polytraumatic injuries. When patients arrived to our institution, 109 (93.2%) received IV antibiotics while still in the Emergency Department. There were 100 (85.5%) patients whom received cefazolin, per protocol, while 17 (14.5%) received an alternative antibiotic (e.g. clindamycin, metronidazole, vancomycin, ampicillin/sulbactam), not including gentamicin or penicillin, 8 (6.8%) of which were due to cephalosporin/penicillin allergy. Patients with allergies were included in the data series, and the alternative antibiotic used in lieu of cefazolin was used to calculate administration time. Summary of patient demographic and injury data is presented in Table 1.

**Table 1:** Patient demographic data.

No. of Patients	117
Age (years)	35.2 ± 13.9 (1 SD)
**Gender**	
Female	29 (24.8%)
Male	88 (75.2%)
BMI (kg/m^2^)	29.5 ± 7.3 (1 SD)
Transportation Time (min.)	23.8 ± 9.1 (1 SD)
**Transportation Method**	
EMS	83 (70.9%)
Police	13 (11.1%)
Transfer	7 (6.0%)
Walk-in	14 (12.0%)
**Injury Statistics**	
**Fracture Location**	
Upper Extremity	36 (30.8%)
Lower Extremity	81 (69.2%)
**Gustilo-Anderson Classification**	
Type I	53 (45.3%)
Type II	25 (21.4%)
Type IIIa	25 (21.4%)
Type IIIb	10 (8.5%)
Type IIIc	4 (3.4%)

Summary of patient demographic data including age ranges and average BMI, as well as injury statistics including site of injury and Gustilo classification

**Fig 1:**
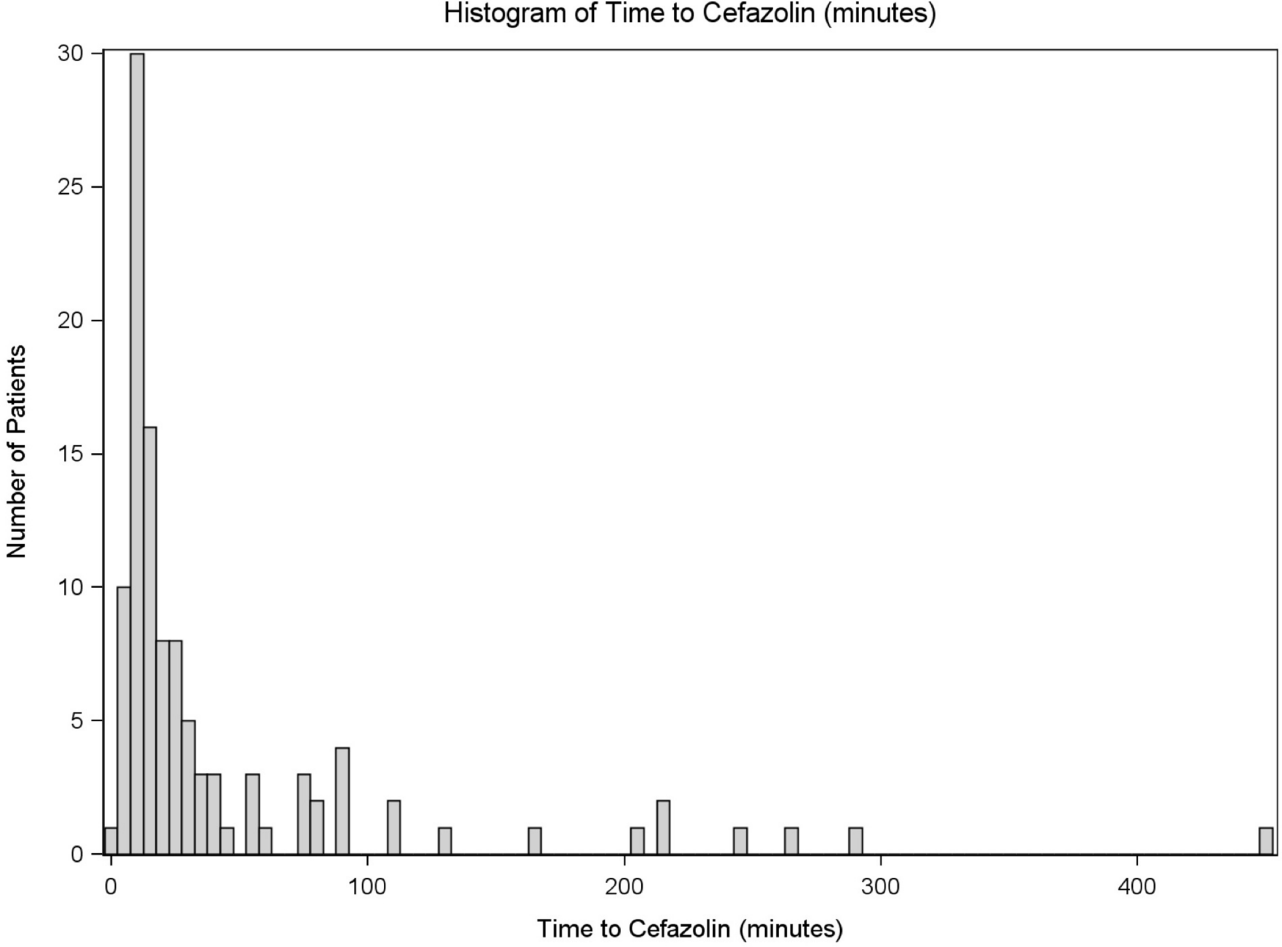
Timing of cefazolin for all patients. Demonstration of the distribution of patients and their associated time to cefazolin administration. Patients are not classified based on open fracture type or triaging group.

Timing of the administration of cefazolin is summarized in Table 2 and illustrated in Fig 1. Cefazolin was given to 109 patients with a median time to administration of 17 minutes with a range of 2 to 448 minutes. Males receive cefazolin at a median of 14 minutes after arrival to the emergency department, while females receive cefazolin at a median of 31 minutes after arrival (P = 0.347). Patients given antibiotics in the emergency department receive cefazolin 15 minutes after arrival while those not given antibiotics in the emergency department receive cefazolin 214 minutes after arrival (P = 0.001). Patients upgraded to a trauma team activation, receive cefazolin 14 minutes after arrival; those not upgraded to trauma, receive cefazolin 53 minutes after arrival (P = <0.0001). Patients with type I fractures received cefazolin 18 minutes after arrival; type II, 19 minutes after arrival; type IIIa, 15 minutes after arrival; type IIIb, 13 minutes after arrival; and type IIIc, 13 minutes after arrival (P = 0.491).

**Table 2:** Summary of timing to cefazolin administration.

*Classification Variable*	*N*	*Mean*	*StdDev*	*Median*	*Interquartile Range*	*Parametric p-Value*	*Parametric Method*	*Non-Parametric p-Value*	*Non-Parametric Method*
Time to Cefazolin by Gender						0.347	t-test	0.011	Wilcoxon
Female	26	55	63	31	15–81				
Male	83	40	71	14	10–30				
Time to Cefazolin by Gustilo Type						0.491	ANOVA	0.532	Kruskal-Wallis
1	50	56	89	18	10–58				
2	25	36	38	19	12–36				
3a	20	31	47	15	9–31				
3b	10	34	64	13	10–22				
3c	4	17	16	13	8–27				
Time to Cefazolin by if Antibiotics given in ER						0.046	t-test	0.001	Wilcoxon
Yes	102	34	49	15	10–31				
No	7	177	151	214	41–245				
Time to Cefazolin by Trauma Team Activation						0.059	t-test	<0.0001	Wilcoxon
Yes	85	37	71	14	10–26				
No	24	67	59	53	28–89				

Summary of the timing to cefazolin administration as compared to variables including gender, Gustilo type, if antibiotics were given in the emergency department and if the patient was upgraded to a trauma team activation.

**Fig 2:**
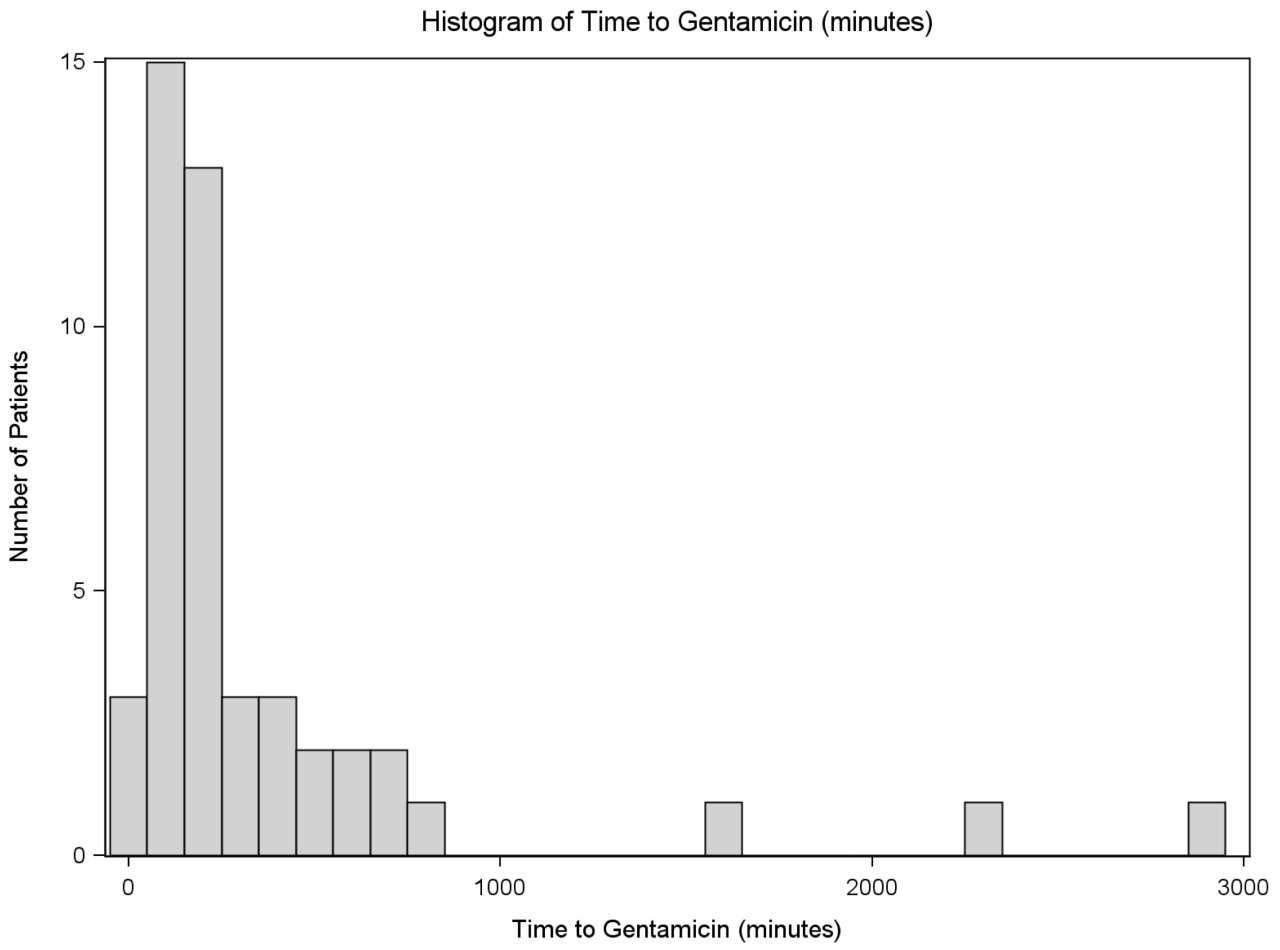
Timing of gentamicin for all patients. Demonstration of the distribution of all patients and their associated time to gentamicin administration. Patients are not classified based on open fracture type or triaging group.

The timing to administration of gentamicin is summarized in Table 3. Gentamicin was administered to 47 of 117 patients a median of 180 minutes after arrival with a range of 28 to 2852 minutes, illustrated in Fig 2. The time to gentamicin administration for females was 208 minutes (median), and for males was 167 minutes (P = 0.189). Out of the 47 patients who received gentamicin, 43 received antibiotics in the emergency department and therefore received gentamicin 175 minutes after arrival. The four patients who did not receive any antibiotics in the emergency department received gentamicin on average 625 minutes after arrival (P = 0.026). Patients who were upgraded to a trauma activation received gentamicin 176 minutes after arrival, while patients that were not activated as a formal trauma received gentamicin 263 minutes after arrival (P = 0.375). Patients with type I fractures received gentamicin 165 minutes after arrival; type II, 188 minutes after arrival; type IIIa, 176 minutes after arrival; type IIIb, 227 minutes after arrival; and type IIIc, 424 minutes after arrival (P = 0.962). In addition to cefazolin and gentamicin, penicillin was given to 5 patients an average of 184 minutes after arrival. Seventeen patients received alternative antibiotics on average 44 minutes after arrival (due to allergy to cefazolin).

**Table 3:** Summary of timing to gentamicin administration.

*Classification Variable*	*N*	*Mean*	*StdDev*	*Median*	*Interquartile Range*	*Parametric p-Value*	*Parametric Method*	*Non-Parametric p-Value*	*Non-Parametric Method*
Time to Gentamicin by Gender						0.189	ANOVA	0.729	Wilcoxon
Female	16	249	177	208	131–354				
Male	31	423	657	167	93–477				
Time to Gentamicin by Gustilo Type						0.962	ANOVA	0.817	Kruskal-Wallis
1	3	271	266	165	74–574				
2	13	337	416	188	104–346				
3a	19	334	629	176	87–233				
3b	10	480	664	227	116–510				
3c	2	424	462	424	97–750				
Time to Gentamicin by if Antibiotics given in ER						0.285	t-test	0.026	Wilcoxon
Yes	43	299	414	175	93–346				
No	4	1078	1199	625	393–1764				
Time to Gentamicin by Trauma Team Activation						0.860	t-test	0.375	Wilcoxon
Yes	43	370	569	176	93–365				
No	4	319	188	263	178–460				

Summary of the timing to gentamicin administration as compared to variables including gender, Gustilo type, if antibiotics were given in the emergency department and if the patient was upgraded to a trauma team activation.

## Discussion

A statistical difference was found between the timing to cefazolin administration in the trauma activation patient group and the non-trauma patient group (14 minutes versus 53 minutes after arrival, P = <0.0001). Refer to Table 4 for detailed breakdown. At our institution, cefazolin is stored in the trauma bay and is provided to all trauma patients immediately if there is clinical suspicion of an open fracture. When patients arrive to the emergency room and are not upgraded to a trauma activation, they are evaluated by the emergency department physicians in a time frame that is less predictable than those who are brought to the trauma bay urgently. Our review of the records reveals that a common delay in cefazolin administration in non-trauma patients occurred between the emergency medicine physician initial evaluation and the time that the order for cefazolin was placed. Additionally, 6 patient charts (accounting for 23% of those assessed by the ER alone) showed that the emergency medicine physician ordered antibiotics after consulting the orthopaedic service. To address this issue, an educational campaign could be implemented with the Emergency Department staff that reiterates the importance of hospital protocols regarding antibiotic administration in open fractures. This formal education has proven effective in reducing time to antibiotics in other institutions [15,16].

**Table 4:** Time to cefazolin partitioned by trauma team activation and gender.

*Variable*	*N*	*Mean*	*StdDev*	*Median*	*Interquartile Range*
**Time to Ancef (minutes)**					
Trauma & Male	68	36	75	13	9–23
No Trauma & Male	15	56	46	41	19–76
Trauma & Female	17	39	52	19	10–36
No Trauma & Female	9	85	75	81	36–89
Total	109	43	69	17	10–40

A significant difference between the timing to cefazolin administration and the timing to gentamicin administration of 163 minutes was found, with the average time to gentamicin administration after arrival being 180 minutes. We expected a discrepancy when comparing delivery times between the two antibiotics because unlike cefazolin, gentamicin is not stored in the emergency department at our institution, but rather is sent from the main hospital pharmacy following a physician’s orders. This is common in almost all institutions due to the variability in dosing (weight-based) which makes standard dose availability in drug storing machines difficult. The 180 minutes to gentamicin administration and 53 minutes to cefazolin administration (when assessed by the ER) after patient arrival places administration beyond the 66 minutes recommended by Lack et al [14].

During our investigation, we found several potential reasons for this delay. First, some patients were transferred out of the emergency department prior to a physician ordering gentamicin, most often when the general surgery team was responsible for placing antibiotic orders for the patients. In other cases, when gentamicin was ordered in the emergency department, no documentation was found that it was actually administered by the nurses. Moreover, gentamicin was often given in the operating room by the anesthesiologist, or rarely, it was ordered postoperatively. Often in these intra-operative cases, the patient arrived in the operating room before the gentamicin was delivered from the hospital pharmacy. In a few cases, the surgeon determined intra-operatively that the open fracture indicated gentamicin, and therefore was given by anesthesia. It has been substantiated that the Gustilo classification be utilized as an intraoperative assessment tool [17,18]; therefore, giving gentamicin in the operating room seems reasonable if a fracture type was upgraded. However, the average time to surgical debridement was 403 minutes, so in cases in which there is a high suspicion for a high-grade fracture based on fracture pattern or obvious soft tissue damage then gentamicin should be ordered promptly prior to debridement.

There were several limitations in this current study. Due to its retrospective design and the relatively small sample size, data was obtained from what was presented in the medical record. Missing information and inconsistencies could contribute a source of error in data collection. Additionally, since the timing of antibiotics was determined retrospectively, common sources of delay could only be speculated. A subgroup analysis was not performed in regards to Gustilo fracture type and gender, and as such these variables may be underpowered. Finally, without examining the true primary endpoint and goal of antibiotic administration (rate of infection), it is beyond the scope of this study to ascertain if this delay results in poorer clinical outcomes in this patient population.

## Conclusion

Patients who arrive to our institution with open fractures and are upgraded as a trauma team activation receive the first antibiotic within an average of 30 minutes of arrival. However, there is room for improvement in the treatment of non-trauma activation patients (which can take up to 1 hour after arrival to administer the first antibiotic) and those requiring gentamicin (which can take up to 5 hours to administer). Ways to improvement include more extensive education and awareness of triaging doctors to open fractures. To better comply with current recommendations for infection prevention institutions should aim to administer antibiotics as rapidly as possible. Further studies with a larger sample size are necessary to validate the results of this study and help identify sources of delay at our institution. A large, prospective study examining the time to antibiotic administration in the emergency department and resultant risk of surgical site infection could further establish more effective institutional protocols.
